# Impact of family doctor system on chronic patients with distinct service utilization patterns

**DOI:** 10.1093/geroni/igaf122.2264

**Published:** 2025-12-31

**Authors:** Guanggang Feng, Lingxue Sun

**Affiliations:** Anhui University of Finance and Economics, Bengbu, Anhui, China (People’s Republic); Shanghai Administration Institute, Shanghai, Shanghai, China (People’s Republic)

## Abstract

This study evaluates the impact of China’s Family Doctor System (FDS) on healthcare utilization and costs among chronic patients using group-based trajectory modeling (GBTM) and difference-in-differences (DID) analysis. Data from 306,464 chronic patients (mean age 60.37, 45.6% female) in City S (2018-2023) was analyzed, with 75.9% enrolled in FDS (intervention) and 24.1% non-enrolled (control). Four service utilization trajectory groups were identified through GBTM: low utilization (18.7%), high utilization (23.5%), high community visits (32.2%), and high hospital visits (25.6%). After propensity score matching (PSM), DID analysis revealed FDS significantly increased primary care visits by 28.3% (p < 0.01) and costs by 19.7% (p < 0.05), while reducing secondary/tertiary hospital visits by 15.2% (p < 0.01) and costs by 12.5% (p < 0.05). Subgroup analysis showed the largest effects among historically high hospital users, with a 21.3% reduction in hospital costs (p < 0.001). The SUEST test confirmed model consistency across trajectory groups (p = 0.68). Ongoing regression results further validate FDS effectiveness in optimizing resource allocation by shifting care to primary settings. Policymakers should strengthen FDS through financial incentives and capacity building to enhance chronic disease management. This research provides empirical evidence for deepening hierarchical medical system reforms by demonstrating FDS’s role in promoting primary care utilization and reducing costly hospital dependency.

